# Identification of risk factors for attempted suicide by self-poisoning and a nomogram to predict self-poisoning suicide

**DOI:** 10.3389/fpubh.2023.1106454

**Published:** 2023-03-08

**Authors:** Wenjing Zheng, Le Gao, Yanna Fan, Chunyan Wang, Yanqing Liu, Fei Tian, Min Yi, Xiaobo Peng, Chunzi Liu

**Affiliations:** ^1^Department of Chemical Poisoning Treatment, Senior Department of Hematology, The Fifth Medical Center of PLA General Hospital, Beijing, China; ^2^Department of Oncology, Senior Department of Oncology, The Fifth Medical Center of PLA General Hospital, Beijing, China; ^3^Department of Radiation Oncology, Senior Department of Oncology, The Fifth Medical Center of PLA General Hospital, Beijing, China; ^4^Institute of Medical Information and Library, Chinese Academy of Medical Sciences and Peking Union Medical College, Beijing, China; ^5^Senior Department of Oncology, The Fifth Medical Center of PLA General Hospital, Beijing, China

**Keywords:** suicide, self-poisoning, prediction model, nomogram, mental health

## Abstract

**Purpose:**

Suicide is a global concern, especially among young people. Suicide prediction models have the potential to make it easier to identify patients who are at a high risk of suicide, but they have very little predictive power when there is a positive value for suicide mortality. Therefore, the aim of the study is to uncover potential risk factors associated with suicide by self-poisoning and further to provide a trustworthy nomogram to predict self-poisoning suicide among poisoned patients.

**Methods:**

This study prospectively enrolled 237 patients who were treated for poisoning at the Fifth Medical Center of PLA General Hospital (Beijing) between May 2021 and May 2022. Patient's basic characteristics, daily activities, mental health status, and history of psychological illnesses were gathered to examine their predictive power for self-poisoning suicide. On developing a prediction model, patients were split 8:2 into a training (*n* = 196) group and a validation (*n* = 41) group at random *via* computer. The training group worked on model development, while the validation group worked on model validation. In this study, the Hosmer and Lemeshow test, accuracy, and area under the curve were the primary evaluation criteria. Shapley Additive exPlanations (SHAP) was determined to evaluate feature importance. To make the prediction model easy for researchers to utilize, it was presented in nomogram format. Two risk groups of patients were identified based on the ideal cut-off value.

**Results:**

Of all poisoned patients, 64.6% committed suicide by self-poisoning. With regard to self-poisoning attempted suicide, multivariate analysis demonstrated that female gender, smoking, generalized anxiety disorder-7 (GAD-7), and beck hopelessness scale-20 (BHS-20) were significant risk factors, whereas married status, relatively higher education level, a sedentary time of 1–3 h per day, higher sport frequency per week, higher monthly income were significant protective features. The nomogram contained each of the aforementioned nine features. In the training group, the area under curve (AUC) of the nomogram was up to 0.938 (0.904–0.972), whereas in the validation group, it reached a maximum of 0.974 (0.937–1.000). Corresponding accuracy rates were up to 0.883 and 0.927, respectively, and the *P*-values for the Hosmer and Lemeshow test were 0.178 and 0.346, respectively. SHAP demonstrated that the top three most important features were BHS-20, GAD-7, and marital status. Based on the best cut-off value of the nomogram (40%), patients in the high-risk group had a nearly six-time larger likelihood of committing suicide by self-poisoning than patients in the low-risk group (88.68 vs. 15.38%, *P* < 0.001). The dynamic nomogram was made available at the following address: https://xiaobo.shinyapps.io/Nomogramselfpoisoningsuicide/.

**Conclusions:**

This study proposes a prediction model to stratify patients at a high risk of suicide by self-poisoning and to guide individual preventive strategies. Patients in the high-risk group require further mental health counseling to alleviate anxiety and hopelessness, healthy lifestyle like quitting smoking and exercising more, and restriction of access to poison and psychiatric drugs.

## Introduction

Suicide is the behavior of deliberately causing one's own death, and it is one of serious causes of death all over the world (1). Suicide accounted for 1.4% of all deaths globally, which meant above 700,000 people died due to suicide every year (2). It should be noted that suicide rates are rising, and while it is not the top cause of death overall, it is the leading cause of death among children and adolescents (1–3). According to the US Centers for Disease Control and Prevention's criteria, suicide really encompasses a number of stages, including ideation, planning, attempt, and completion. Up to 9.2–13.0% of youth may have suicidal thoughts, and 4.8–7% of those reported at least one suicide attempt in the previous year (4–6). Suicide is a global public health problem causing a huge economic, social, and psychological burden on individuals, families, and communities.

It is extremely helpful to identify suicide risk factors in order to direct preventive and treatment strategies. Currently, a number of variables haven been shown to be relevant to suicide ideations or attempts, such as sex (5), family integrity (5), feeling meaningless (5), depression (5), self-esteem (5), hopelessness (5), stressful life events (5), social support, high physical and mental exhaustion, prior suicide attempt, sleep disturbances, loneliness (7), alcohol consumption (7), and mental health difficulties (8). More recently, a study elucidated that age, sex, residence, socioeconomical standard, and occupation were significantly associated with self-poisoning suicide (9). Although these factors could have some references for physicians to screen individuals at a high risk of self-poisoning, the above any common risk factors were present in individuals who do not directly involve in suicide, causing important questions about how well-known risk factors can identify those who are truly at risk for suicide (10). In addition, a thorough meta-analysis of more than 3,400 risk variables for suicide found that all of them were insufficient and inaccurate contributors to suicide, which might in part be attributable to the methodological imitations of the literature (11).

Several studies have developed models to predict suicide ideation among cancer patients (12) and adolescent after the COVID-19 pandemic (13), and to predict suicide attempt among depressed population (14) and patients with major depressive disorder (15). The predictive validity associated with a positive value for suicide mortality was extremely low, indicating that the current models offer limited practical utility in predicting suicide, even though suicide prediction models have the potential to improve the identification of patients at a high risk of suicide (10). Additionally, a model to specifically predict self-poisoning suicide is still unavailable among poisoned patients.

Nomogram is combined as a magnificent visual depiction of a discrimination procedure from a predictive regression model (16), and it has already been widely used as a prognostic tool in the field of oncology and medicine (16, 17). Nomogram, which has the advantage of rapid computation *via* user-friendly graphical or digital interfaces, embraces increased accuracy and more easily understood prognoses in comparison to conventional staging, is able to generate a personalized risk of a clinical event by incorporating various prognostic and determinant features (16, 17). In addition, a dynamic nomogram can be displayed directly on the internet, making it accessible to users whenever they have internet-connected electronic devices (18).

Therefore, the purpose of this study was to identify risk variables for self-poisoning suicide as well as to suggest an accurate nomogram for predicting self-poisoning suicide.

## Patients and methods

### Patients

In this study, 267 patients who were admitted to the Fifth Medical Center of PLA General Hospital (Beijing) for poisoning treatment between May 2021 and May 2022 were prospectively examined. Basic patient characteristics, lifestyle, poison type, length of stay, medical costs, mental health condition, and history of psychiatric illnesses were collected for this investigation. Patients were included if he/she was admitted to our department due to poisoning. Patients were excluded if he/she was (1) unwilling to take part in the survey, (2) unconsciousness, (3) unable to cooperate with doctors due to any other reasons, and (4) dead during hospitalization.

**Figure 1:**
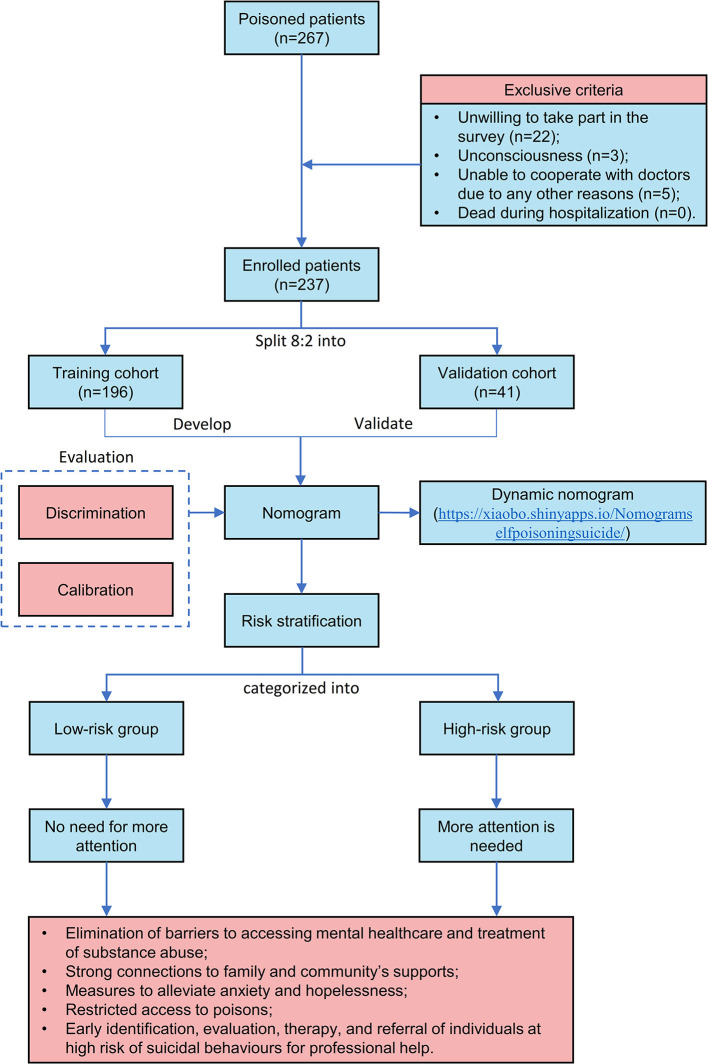
Patient's flowchart and creation of a nomogram.

Based on the exclusive and inclusive criteria, a total of 237 patients were enrolled for analysis in the study. Patient's flowchart is depicted in Figure 1. The ratio of 80:20 was used to randomly divide all patients into two groups. Model construction then took place in the training group (*n* = 196) and model validation in the validation group (*n* = 41). The Ethics Committee of the Fifth Medical Center of PLA General Hospital approved the study protocol (No. KY-2021-12-34-1). Data were anonymously analyzed and informed written consent was obtained from all patients.

### Data collection

The following variables were gathered for this study: (1) basic characteristics (age, gender, marital status, education level, and residence), (2) lifestyle (bland diet, greasy food, smoking, drinking, sedentary time per day, sport frequency per week, and monthly income), (3) mental health status (anxiety, depression, self-esteem, beck hopelessness, and social support), and (4) history of psychological disease (history of depression and history of psychiatry disease). Patient's lifestyle was self-reported and mental health status was evaluated using five scales *via* face-to-face interview. Anxiety was evaluated using the generalized anxiety disorder-7 (GAD-7) (19). GAD-7 score is ranged from 0 to 21, and a higher score indicates a more serious anxious status. Depression was evaluated using the patient health questionnaire-9 (PHQ-9) (19). PHQ-9 has a score of 0 to 27 with higher scores indicating severer depressive conditions. Patient's self-esteem was assessed using the self-esteem scale-10 (SES-10) (20). SES-10 was widely sued to assess the individual's overall feelings about self-worth and self-acceptance. A higher SES-10 score indicates better self-esteem. Patient's hopelessness status was measured using the Beck hopelessness scale-20 (BHS-20) (21). Higher scores on the BHS-20 scale, which ranges from 0 to 20, indicate greater hopelessness. Patient's social support status was evaluated using the social support questionnaire-10 (SSQ-10) (22). Better social support is indicated by a higher SSQ-10 score.

In addition, type of poison, length of stay, and medical expense were also collected for analysis. In the study, type of poison was categorized into four main categories, including sleeping pills, pesticides, psychotropic drugs, and others. Length of stay was the time interval between patient's admission and discharge date. Medical expense was the total of expense that used to the treatment of poisoning in the hospital. In the study, self-poisoning was regarded as the positive event, and patients who admitted to our medical center not due to self-poisoning were served as the negative control. Poisoned patients who were not self-poisoning were served as the healthy controls, because those patients admitted to our medical center usually due to accidental poisoning.

### Nomogram development and validation

Multivariate analysis was utilized to build the nomogram and discover variables linked to self-poisoning suicide. A nomogram was created using variables that reached statistical significance. The nomogram was displayed *via* the “regplot” package. Validation of the nomogram was conducted using discrimination and calibration. Discrimination was the capacity to discriminate patients with and without the positive event (23, 24), and the metrics that used to evaluate discrimination mainly included area under curve (AUC) and discrimination slope. Calibration was defined as the consistency of the anticipated and observed probability of the positive event (23, 24), and the metrics mainly included Brier score, Brier_scaled_ score, and Hosmer and Lemeshow test. In addition, nomogram's accuracy, specificity, sensitivity, negative predictive value (NPV), positive predictive value (PPV), precision, recall, and Youden index.

### Feature importance analysis

For clinical applicability, Shaley Additive exPlanation (SHAP) was used to interpret feature contributions. On explaining SHAP, we adopted the following formula, and in the formula *g* is the interpretation model, *M* is the number of input parameters, ϕ_0_ is a constant, and ϕ_*j*_ is the attribution value (Shapley value) of each model parameter.


$$\begin{array}{l} g\left(z^{{}^{\prime }}\right)=\phi_{0}+\overset{M}{\underset{j=1}{\sum }}\phi_{j}{Z^{{}^{\prime }}}_{j} \end{array}$$

### Statistical analysis

In the study, quantitative features were presented as means with standard deviation, and qualitative features were summarized as proportion. Comparison between quantitative features were evaluated using *t*-test, and qualitative features were assessed using Chi-square test and continuous adjusted Chi-square test. The potential of risk variables to predict suicide was tested using simple and multiple logistic regression analysis. Odd ratios (ORs) and corresponding 95% confident interval were also calculated in the study. The ideal cut-off value was thought to be the average threshold between the training and validation cohorts. All patients were categorized into two groups, i.e., a low-risk group and a high-risk group. The anticipated suicide probability for patients in the low-risk group was less than the ideal cut-off value, but the predicted suicide probability for patients in the high-risk group was higher than the ideal cut-off value. A probability level of 0.05 (two-sided) was conducted for all statistical analyses and R programming language (version 4.1.2) was used for descriptive analyses and data visualization.

## Results

### Patient's basic characteristics and clinical features

A total of 237 patients were enrolled for analysis in the study with a mean age of 33.33 ± 15.83 years. The majority of patients were female (55.3%), married (48.5%), primary education level (37.1%), and lived in city (71.7%). Regarding dietary preference, 54.4% patients intended to bland diet and 84.4% patients had not an inclination to greasy food. The number of patients who were smoking and drinking accounted for 32.1 and 18.1%, respectively. Regarding sport habit, only 33.8% patients did exercise for three or above times each week, and also 33.8% patients had a sedentary time of three or above hours each day. A multitude of patients were in a relatively low-income status since up to 50.2% had a monthly income of <3,000¥. Type of poison mainly included sleeping pills (21.9%), pesticides (25.3%), and psychotropic drugs (10.6%). The mean length of stay was 12.36 days and the mean medical expense was 44,822.25¥. Table 1 provides a summary of further information on the patient's mental health and previous psychological illnesses. Of all poisoned patients admitted to our department, 64.6% were due to suicide by self-poisoning.

**Table 1:** Patient's basic characteristics, living habit, and mental health status.

**Characteristics**	**Overall**
*n*	237
Age [mean (SD), years]	33.33 (15.83)
**Gender (%)**	
Male	106 (44.7)
Female	131 (55.3)
**Marital status (%)**	
Single	93 (39.2)
Dating	18 (7.6)
Married	115 (48.5)
Divorced or widowed	11 (4.6)
**Education level (%)**	
Primary	88 (37.1)
High school	55 (23.2)
University	84 (35.4)
Graduate	10 (4.2)
**Residence (%)**	
City	170 (71.7)
Countryside	67 (28.3)
**Bland diet (%)**	
Yes	129 (54.4)
No	108 (45.6)
**Greasy food (%)**	
Yes	37 (15.6)
No	200 (84.4)
**Smoking (%)**	
Yes	76 (32.1)
No	161 (67.9)
**Drinking (%)**	
Yes	43 (18.1)
No	194 (81.9)
**Sedentary time per day (hours, %)**	
< 1	64 (27.0)
≥1 and <3	93 (39.2)
≥3 and <6	40 (16.9)
≥6	40 (16.9)
**Sport frequency per week (%)**	
0	55 (23.2)
1–2	102 (43.0)
3–5	49 (20.7)
>5	31 (13.1)
**Monthly income (%)**	
<3,000	119 (50.2)
≥3,000 and <6,000	73 (30.8)
≥6,000 and <9,000	21 (8.9)
≥9,000	24 (10.1)
GAD-7 [mean (SD)]	8.71 (6.83)
PHQ-9 [mean (SD)]	11.40 (8.73)
SES-10 [mean (SD)]	26.49 (6.14)
BHS-20 [mean (SD)]	9.42 (4.42)
SSQ-10 [mean (SD)]	34.93 (10.45)
**History of depression (%)**	
Yes	82 (34.6)
No	155 (65.4)
**History of psychiatry disease (%)**	
Yes	43 (18.1)
No	194 (81.9)
**Type of poison**	
Sleeping pills	52 (21.9)
Pesticides	60 (25.3)
Psychotropic drugs	25 (10.6)
Others	100 (42.2)
Length of stay [mean (SD), days]	12.36 (9.41)
Medical expense [mean (SD), ¥]	44,822.25 (37,700.11)
**Suicide**	
Yes	153 (64.6)
No	84 (35.4)

SD, standard deviation; GAD-7, generalized anxiety disorder-7; PHQ-9, patient health questionnaire-9; SES-10, self-esteem scale-10; BHS-20, beck hopelessness scale-20; SSQ-10, social support questionnaire-10.

### A comparison analysis based on the presence of suicide

Between patients who committed suicide and those who did not, significant differences in gender (*P* = 0.006), age (*P* < 0.001), marital status (*P* < 0.001), greasy food (*P* = 0.014), sport frequency per week (*P* = 0.001), monthly income (*P* = 0.001), GAD-7 (*P* < 0.001), PHQ-9 (*P* < 0.001), SES-10 (*P* < 0.001), BHS-20 (*P* < 0.001), SSQ-10 (*P* < 0.001), history of depression (*P* < 0.001), and history of psychiatry disease (*P* = 0.016) (Table 2). More explicitly, self-poisoning suicide patients tended to be female, younger, single, and consuming more greasy food. In addition, they had lower sport frequency per week, lower monthly income, higher GAD-7 score, higher PHQ-9 score, higher BHS-20 score, lower SES-10 score, lower SSQ-10 score, and higher rates of history of depression and psychiatry diseases compared to poisoned patients without suicide. The aforementioned findings showed that self-poisoning suicide patients had unfavorable living conditions, financial challenges, and poor psychological health.

**Table 2:** A comparison between patients with and without suicide in the training group.

**Characteristics**	**Overall**	**Suicide**		***P*** **-value**
		**Yes**	**No**	
*n*	196	69	127	
Gender (male/female, %)	81/115 (41.3/58.7)	38/31 (55.1/44.9)	43/84 (33.9/66.1)	0.006
Age [mean (SD)]	32.91 (15.54)	39.97 (13.70)	29.08 (15.17)	<0.001
Marital status (%)				<0.001
Single	77 (39.3)	10 (14.5)	67 (52.8)	
Dating	15 (7.7)	6 (8.7)	9 (7.1)	
Married	93 (47.4)	50 (72.5)	43 (33.9)	
Divorced or widowed	11 (5.6)	3 (4.3)	8 (6.3)	
Education level (%)				0.153
Primary	72 (36.7)	19 (27.5)	53 (41.7)	
High school	47 (24.0)	20 (29.0)	27 (21.3)	
University	68 (34.7)	25 (36.2)	43 (33.9)	
Graduate	9 (4.6)	5 (7.2)	4 (3.1)	
Residence (city/countryside, %)	140/56 (71.4/28.6)	51/18 (73.9/26.1)	89/38 (70.1/29.9)	0.688
Bland diet (yes/no, %)	103/93 (52.6/47.4)	41/28 (59.4/40.6)	62/65 (48.8/51.2)	0.204
Greasy food (yes/no, %)	33/163 (16.8/83.2)	5/64 (7.2/92.8)	28/99 (22.0/78.0)	0.014
Smoking (yes/no, %)	67/129 (34.2/65.8)	20/49 (29.0/71.0)	47/80 (37.0/63.0)	0.330
Drinking (yes/no, %)	38/158 (19.4/80.6)	12/57 (17.4/82.6)	26/101 (20.5/79.5)	0.740
Sedentary time per day (hours, %)				0.137
< 1	52 (26.5)	20 (29.0)	32 (25.2)	
≥1 and <3	72 (36.7)	31 (44.9)	41 (32.3)	
≥3 and <6	38 (19.4)	10 (14.5)	28 (22.0)	
≥6	34 (17.3)	8 (11.6)	26 (20.5)	
Sport frequency per week (%)				0.001
0	52 (26.5)	12 (17.4)	40 (31.5)	
1–2	80 (40.8)	28 (40.6)	52 (40.9)	
3–5	39 (19.9)	12 (17.4)	27 (21.3)	
>5	25 (12.8)	17 (24.6)	8 (6.3)	
Monthly income (%)				0.001
<3,000	98 (50.0)	23 (33.3)	75 (59.1)	
≥3,000 and <6,000	55 (28.1)	25 (36.2)	30 (23.6)	
≥6,000 and <9,000	21 (10.7)	7 (10.1)	14 (11.0)	
≥9,000	22 (11.2)	14 (20.3)	8 (6.3)	
GAD-7 [mean (SD)]	8.85 (6.87)	3.97 (5.37)	11.50 (6.12)	< 0.001
PHQ-9 [mean (SD)]	11.57 (8.75)	5.91 (7.23)	14.64 (7.95)	<0.001
SES-10 [mean (SD)]	26.33 (6.22)	29.52 (5.16)	24.60 (6.07)	<0.001
BHS-20 [mean (SD)]	9.47 (4.44)	6.20 (3.25)	11.25 (3.97)	<0.001
SSQ-10 [mean (SD)]	34.69 (10.39)	38.75 (11.07)	32.48 (9.32)	<0.001
History of depression (yes/no, %)	67/129 (34.2/65.8)	7/62 (10.1/89.9)	60/67 (47.2/52.8)	<0.001
History of psychiatry disease (yes/no, %)	29/167 (14.8/85.2)	4/65 (5.8/94.2)	25/102 (19.7/80.3)	0.016

SD, standard deviation; GAD-7, generalized anxiety disorder-7; PHQ-9, patient health questionnaire-9; SES-10, self-esteem scale-10; BHS-20, beck hopelessness scale-20; SSQ-10, social support questionnaire-10.

### Development of the nomogram

**Figure 2:**
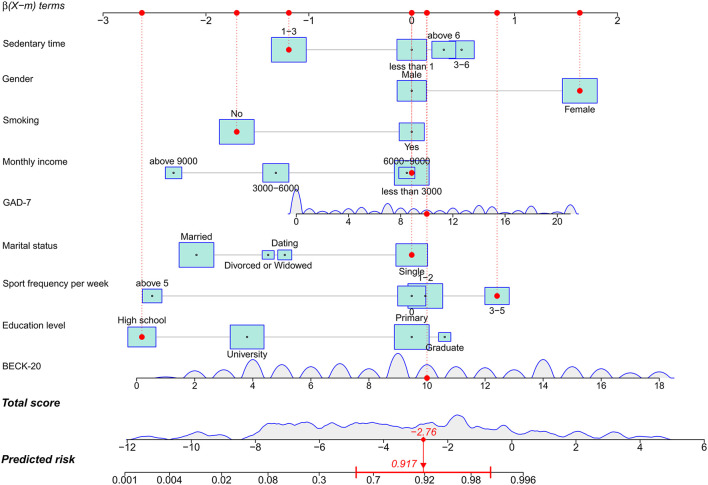
A nomogram to predict risk of self-poisoning. The red dot in each characteristic in the supplied case showed the case's current condition. For instance, the red dot was placed at the “No” box because the case was not a smoker. To determine a unique score for the feature, a line was drawn upward from the box to the score axis. All nine features were combined to create the final score (−2.76). We were able to determine the patients' final suicide risk (91.7%) by drawing a line downward to the projected risk axis.

Multivariate analysis demonstrated that female gender (*P* = 0.001), smoking (*P* = 0.024), GAD-7 (*P* = 0.007), and BHS-20 (*P* = 0.007) were significant risk factors for suicide, while married status (*P* = 0.048), relatively higher education level (*P* = 0.003), lower sedentary time (*P* = 0.035), higher sport frequency per week (*P* = 0.007), higher monthly income (*P* = 0.032) were significant protective factors for suicide (Table 3). Therefore, the nomogram contained all nine of the aforementioned features (Figure 2). In the nomogram, a case was shown to depict how to use the nomogram. Based on the nomogram, individual prediction of suicide could be achieved. To promote clinical application, the dynamic nomogram was implemented at: https://xiaobo.shinyapps.io/Nomogramselfpoisoningsuicide/. Users can choose parameters on the left side by clicking the link. Users can acquire the risk of suicide in the dynamic nomogram on the right after selecting items for each parameter by clicking “Predict” at the left bottom of the page. In addition, graphical summary, numerical summary, and model summary are all provided in the dynamic nomogram.

**Table 3:** Selection of nomogram predictor based on the univariate and multivariate analyses in the training group.

**Characteristics**	**Univariate analysis**		**Multivariate analysis**	
	**OR 95% CI**	***P*** **-value**	**OR 95% CI**	***P*** **-value**
**Gender**				
Male	Reference		Reference	
Female	2.39 (1.31–4.36)	0.004	8.85 (2.31–33.88)	0.001
Age	0.95 (0.93–0.97)	0.000	0.96 (0.91–1.02)	0.206
**Marital status**				
Single	Reference		Reference	
Dating	0.22 (0.07–0.76)	0.017	0.29 (0.04–2.28)	0.238
Married	0.13 (0.06–0.28)	0.000	0.16 (0.03–0.98)	0.048
Divorced or widowed	0.40 (0.09–1.76)	0.224	0.62 (0.02–17.02)	0.779
**Education level**				
Primary	Reference		Reference	
High school	0.48 (0.22–1.06)	0.068	0.06 (0.01–0.39)	0.003
University	0.62 (0.30–1.27)	0.188	0.14 (0.02–1.00)	0.050
Graduate	0.29 (0.07–1.18)	0.084	0.75 (0.02–25.94)	0.876
**Residence**				
City	Reference		Reference	
Countryside	1.21 (0.63–2.34)	0.571	3.10 (0.74–13.01)	0.123
**Bland diet**				
Yes	0.65 (0.36–1.18)	0.157	2.80 (0.67–11.75)	0.160
No	Reference		Reference	
**Greasy food**				
Yes	3.62 (1.33–9.86)	0.012	2.91 (0.44–19.02)	0.265
No	Reference		Reference	
**Smoking**				
Yes	Reference		Reference	
No	0.69 (0.37–1.31)	0.259	0.20 (0.05–0.81)	0.024
**Drinking**				
Yes	Reference		Reference	
No	0.82 (0.38–1.74)	0.603	0.58 (0.11–3.14)	0.527
**Sedentary time per day (hours)**				
<1	Reference		Reference	
≥1 and < 3	0.83 (0.40–1.71)	0.608	0.18 (0.04–0.89)	0.035
≥3 and < 6	1.75 (0.70–4.36)	0.230	1.19 (0.18–7.74)	0.858
≥6	2.03 (0.77–5.36)	0.152	1.61 (0.29–8.87)	0.583
**Sport frequency per week**				
0	Reference		Reference	
1–2	0.56 (0.25–1.23)	0.148	1.58 (0.33–7.46)	0.564
3–5	0.68 (0.26–1.72)	0.411	4.66 (0.75–28.97)	0.099
>5	0.14 (0.05–0.41)	0.000	0.08 (0.01–0.49)	0.007
<3,000	Reference		Reference	
≥3,000 and < 6,000	0.37 (0.18–0.75)	0.006	0.21 (0.05–0.88)	0.032
≥6,000 and < 9,000	0.61 (0.22–1.70)	0.348	1.53 (0.24–9.78)	0.653
≥9,000	0.18 (0.07–0.47)	0.001	0.07 (0.01–0.61)	0.015
GAD-7 [mean (SD)]	1.24 (1.16–1.32)	0.000	1.28 (1.07–1.53)	0.007
PHQ-9 [mean (SD)]	1.16 (1.10–1.21)	0.000	0.88 (0.74–1.03)	0.106
SES-10 [mean (SD)]	0.86 (0.81–0.91)	0.000	1.01 (0.88–1.17)	0.853
BHS-20 [mean (SD)]	1.42 (1.28–1.58)	0.000	1.36 (1.09–1.70)	0.007
SSQ-10 [mean (SD)]	0.94 (0.91–0.97)	0.000	0.99 (0.92–1.06)	0.770
**History of depression**				
Yes	Reference		Reference	
No	0.13 (0.05–0.30)	0.000	0.67 (0.14–3.24)	0.620
**History of psychiatry disease**				
Yes	Reference		Reference	
No	0.25 (0.08–0.75)	0.014	0.77 (0.11–5.44)	0.791

OR, odds ratio; CI, confident interval; GAD-7, generalized anxiety disorder-7; PHQ-9, patient health questionnaire-9; SES-10, self-esteem scale-10; BHS-20, beck hopelessness scale-20; SSQ-10, social support questionnaire-10.

### Validation of the nomogram

**Figure 3:**
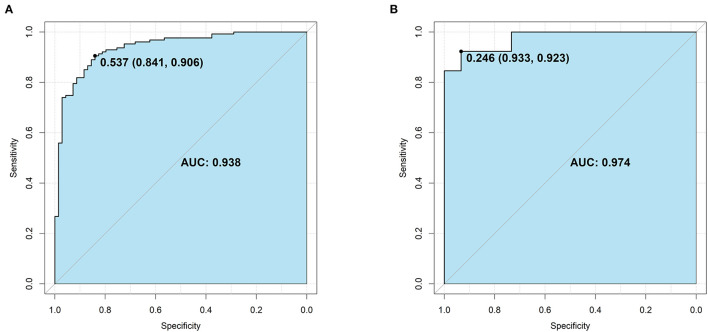
Area under the curve (AUC) for the nomogram. **(A)** The training group. **(B)** The validation group. The light blue indicates the AUC, and its value and the optimal cut-off were provided.

**Figure 4:**
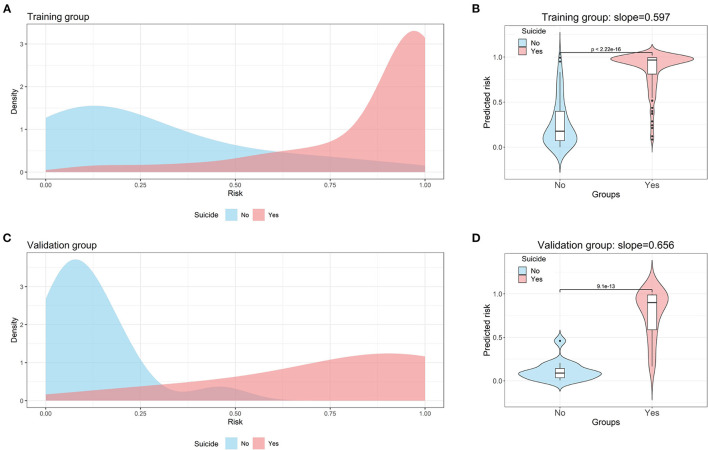
Probability curve and discrimination slope for the nomogram. **(A)** Probability curve for the nomogram in the training group. **(B)** Discrimination slope for the nomogram in the training group. **(C)** Probability curve for the nomogram in the validation group. **(D)** Discrimination slope for the nomogram in the validation group. The light blue indicates patients without suicide. The light red indicates patients with self-poisoning. A large separation between patients with and without suicide was observed in both training and validation groups.

**Figure 5:**
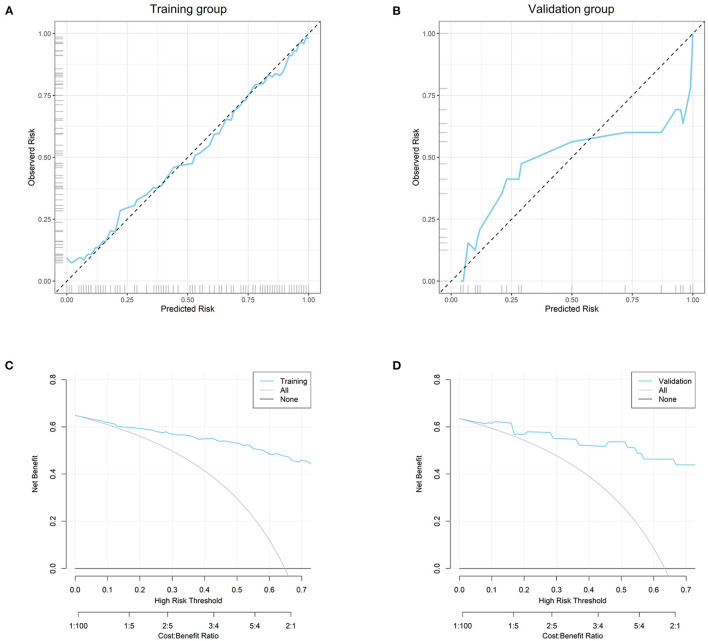
Calibrating evaluation and clinical usefulness of the nomogram. **(A)** Calibration curve in the training group; **(B)** calibration curve in the validation group; **(C)** decision curve analysis in the training group; **(D)** decision curve analysis in the validation group.

The area under curve (AUC) of the nomogram was up to 0.938 (0.904–0.972) in the training group (Figure 3A) and 0.974 (0.937–1.000) in the validation group (Figure 3B). Probability curve showed a large separation between patients with and without suicide in the training group (Figure 4A), indicating favorable discrimination, and it was confirmed by discrimination slope with a value of 0.597 (95% CI: 0.527–0.648, Figure 4B). Probability curve of the validation group is deployed in Figure 4C, and it also showed large separation. The discrimination slope was up to 0.656 (95% CI: 0.533–0.767) in the validation group (Figure 4D). Figures 5A, B demonstrated good consistency between predicted and observed probability in both the training and validation groups. Clinical usefulness was also very favorable in the training (Figure 5C) and validation (Figure 5D) groups as shown by decision analysis curves. Accuracy rates in the two groups were up to 0.883 and 0.927, respectively, and *P*-values of the Hosmer and Lemeshow test were 0.178 and 0.346, respectively. More metrics of predictive evaluation are summarized in Table 4. The aforementioned findings showed that the nomogram was clinically helpful and had outstanding discriminative and calibrating abilities.

**Table 4:** Predictive measures of the nomogram in the training and validation groups.

**Predictive measures**	**Training group**	**Validation group**
AUC (95% CI)	0.938 (0.904–0.972)	0.974 (0.937–1.000)
Discrimination slope (95% CI)	0.597 (0.527–0.648)	0.656 (0.533–0.767)
Threshold	0.537	0.246
Specificity	0.841	0.933
Sensitivity	0.906	0.923
Accuracy	0.883	0.927
NPV	0.829	0.875
PPV	0.913	0.960
Precision	0.913	0.960
Recall	0.906	0.923
Youden	1.746	1.856
Brier score	0.091	0.089
Brier_scaled_ score	0.603	0.618
Hosmer and Lemeshow test	0.178	0.346

AUC, area under the curve; CI, confident interval; NPV, negative predictive value; PPV, positive predictive value.

**Figure 6:**
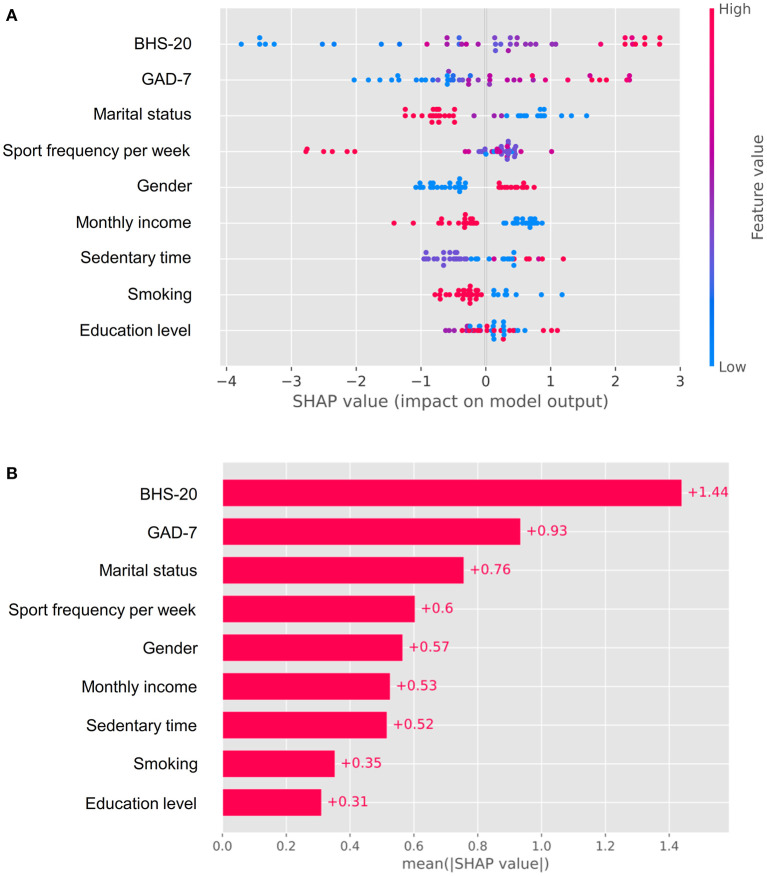
Feature importance analysis. **(A)** Bees warm plot; **(B)** bar plot based on SHAP value.

Additionally, feature importance analysis was employed using SHAP, and it identified that the top three important variables were BHS-20, GAD-7, and marital status (Figure 6), indicating that married status, and measures to alleviate anxiety and hopefulness were considerably beneficial to prevent suicide by self-poisoning.

### Classification of patients at different risk probability of suicide

The average threshold of the training (53.7%) and validation (24.6%) cohorts was regarded as the optimal cut-off value (40%). Based on the best cut-off value of the nomogram, patients in the high-risk group had a nearly 6-time larger likelihood of committing suicide by self-poisoning than patients in the low-risk group (88.68 vs. 15.38%, *P* < 0.001) (Table 5). It proved that effective separation based on risk classification was accomplished.

**Table 5:** Patients stratified by risk group based on the optimal threshold of the nomogram.

**Groups**	**Patients**	**Probability**		***P*** **-value^a^**
		**Predicted**	**Actual**	
Low risk (<40.00%)	78	14.11%	15.38% (12/78)	<0.001
High risk (≥40.00%)	159	86.63%	88.68% (141/159)	

a Indicates a comparison of actual probability between the low- and high-risk groups.

## Discussion

### Main findings

In order to stratify patients at various risk levels for self-poisoning suicide, this study presented a nomogram, and nine features were added to the nomogram for analysis and model building. The predictive performance of the nomogram showed excellent effectiveness of prediction based on the AUC, accuracy rates, and the Hosmer and Lemeshow test. For instance, the AUC value could be up to 0.974 and accuracy rate was 0.927 in the validation group, whereas other studies showed the AUC value ranged from 0.715 to 0.860 in nomograms to suicide ideation among cancer patients (12) and adolescent after the COVID-19 pandemic (13), and to predict suicide attempt among depressed population (14) and patients with major depressive disorder (15).

Additionally, risk stratification was used in the study to conduct personalized medication, and patients in the high-risk group had a nearly 6-time greater chance of committing suicide by self-poisoning than patients in the low-risk group. As a result, this nomogram was useful for identifying those who were at a high risk of self-poisoning suicide. Feature importance analysis demonstrated that the top three important variables were BHS-20, GAD-7, and marital status. Thus, more attention such as effectively preventive strategies in terms of their mental health should be paid to patients in the high-risk group.

### Risk factors associated with suicide behaviors

Studies have shown that a series of variables were associated with suicide attempts, such as sex, grade, residence, family integrity, feeling meaningless in life, depression, bullying perpetrator, autonomy parenting, self-esteem, hopelessness, and stressful life events (5). Physical abuse in childhood, family history of substance misuse, and criminal convictions among family members might also play a role in affecting attempted suicide (25). However, these features were investigated among general adolescents but were not especially for predicting self-poisoning suicide. More recently, a study elucidated that age, sex, residence, socioeconomical standard, and occupation were significantly associated with self-poisoning (9). In addition, a small size analysis pointed out that body mass index, gender, age, and a history of neuropsychiatric disorders were significantly associated with self-poisoning (26). Another study also concluded that pre-existing psychiatric disorder, prior suicide-related behavior, and access to psychiatric medication were also associated with hospitalization due to self-poisoning (27). Among suicidal self-poisoning patients, underlying psychiatry disorders, substance use, and ingestion of neuroleptics or antidepressants were significantly with recurrent suicide (28). In the present study, multivariate analysis also demonstrated that female gender, smoking, depression, and hopelessness were significant risk factors for self-poisoning attempted suicide, while married status, relatively higher education level, lower sedentary time, higher sport frequency per week, higher monthly income were significant protective features for self-poisoning suicide. According to the findings, various actions to stop smoking, treat mental health issues, promote healthy lifestyles, and raise economic standards would be greatly beneficial to avoid suicide by self-poisoning.

### Prediction models of suicide managements

Several prediction models have been developed, according to the literature that is currently accessible, to guide management among self-poisoning patients. For instance, researchers created a nomogram with six features, including age, white cells, albumin, cholinesterase, blood pH, and lactic acid levels, for the bedside assessment of patients with acute organophosphorus poisoning (29). The AUC of the nomogram was very favorable with a value of 0.875 in the derivation group and 0.855 in the validation group. Despite drawing a calibration plot, the calibrating ability was not quantitatively evaluated in the study. This model may be a good tool to identify a high risk of acute organophosphorus poisoning among self-poisoning patients. More recently, Zelkowitz et al. (30) developed a classification and regression tree model for 30 days after psychiatric hospital discharge among female and male patients who attempted suicide, respectively. Different significant factors of non-fatal suicide among patients who were males and women were discussed in this article. Among women patients, history of self-poisoning, substance-related disorders, and eating disorders were important predictors, while as for men patients, self-poisoning, substance-related disorders, and severe stress reactions were strong predictors.

Additionally, a number of studies have developed nomograms to predict suicide ideation among cancer patients (12) and adolescent after the COVID-19 pandemic (13), and to predict suicide attempt among depressed population (14) and patients with major depressive disorder (15). The AUC of those nomograms ranged from 0.715 to 0.860. To the author's knowledge, this study was the first to propose a nomogram to predict the risk of developing self-poisoning suicide. Nine features were included in the nomogram for analysis and model development, with the majority of the nine features being demographic information about the patient, such as gender, marital status, and level of education, as well as information about their lifestyles, such as smoking and exercise, and their mental health, such as anxiety and hopelessness, all of which were generally available. AUC of the nomogram, which indicate outstanding prediction performance, could reach as high as 0.974.

### Approaches to prevent self-poisoning suicide

Identification of risk and protective factors is a crucial step in developing effective suicide prevention methods since it can be used to choose the right interventions and how to carry them out. In these situations, risk factors serve as markers of whether a person or society has a propensity for suicide, hence the development of prediction models is necessary (31). Additionally, a prediction model was developed in the current study to categorize patients according to their risk of suicide.

Because patients in the high-risk group had a nearly six-time greater likelihood of committing suicide by self-poisoning than those in the low-risk group, this model was effective at identifying risk categories. To reduce the risk of suicide at the individual level as much as possible, patients in the high-risk category require extra care while the entire population needs to be the focus at the same time. From an individual standpoint, it will be highly advantageous to promote a healthy lifestyle, treat mental illnesses, and establish solid relationships with families and social communities. These steps should be taken from a sociocultural perspective, including removing obstacles to mental healthcare and drug rehab, reducing media exposure to suicidal behavior and the influence of those who have committed suicide, and limiting access to fatal substances (32, 33); The need for early detection, assessment, therapy, and referral of people at risk of suicide behavior to professionals is emphasized at the national level. Overall, as no single strategy clearly outperforms the others, combinations of evidence-based preventative strategies at the individual and population level may be more beneficial (34). In addition, some measure to keep individuals in married status would be helpful since marital status was one of the top most important features in this study.

### Limitations

There are still a few issues with the study. Firstly, because we can only get data from patients who are still alive, the study may pose a risk of survival bias. Patients who had poisoned themselves and died at home or on the way to hospitals were not accessible to us. Additionally, some suicide attempts could be mistakenly labeled as accidents, making selection bias harder to prevent. Secondly, several ambiguous or conflicting definitions of suicide have complicated international comparisons and impeded development in the area of theory and research (35). Nevertheless, we were able to some extent avoid selection bias when recording occurrences of suicide attempts because we employed the WHO definition in this study. Thirdly, even though the study's constructed model performed well in terms of making predictions, it still requires thorough external validation in a sizable prospective population.

## Conclusions

This study proposes a prediction model to stratify patients at a high risk of suicide by self-poisoning so that individual preventive strategies can be timely performed. Patients in the high-risk group need more healthcare guidance, including education of health lifestyle such as quitting smoking and doing more exercise, restricted access to poison and psychotropic substances, and alleviation of anxiety and hopelessness.

## Data availability statement

The raw data supporting the conclusions of this article will be made available by the authors, without undue reservation.

## Ethics statement

The Ethics Committee of the Fifth Medical Center of PLA General Hospital approved the study protocol (No. KY-2021-12-34-1). Data were anonymously analyzed and informed written consent was obtained from all patients. The Ethics Committee waived the requirement of written informed consent for participation.

## Author contributions

WZ, LG, and CW conceived and designed this study together. WZ and YL undertook the data analysis, results interpretation, and manuscript preparation. CL and XP performed supervision. All authors read and approved the final manuscript.
