# Supplementary material for: Identification of risk factors for attempted suicide by self-poisoning and a nomogram to predict self-poisoning suicide
Source: Front Public Health. 2023 Mar 8;11:1106454. doi: 10.3389/fpubh.2023.1106454 (PMC10031109; doi:10.3389/fpubh.2023.1106454)

**An introduction on the use of the dynamic nomogram**

**1. Screenshot of the dynamic nomogram**

In the left of the screenshot, model parameters, including nine clinical characteristics, are shown in the panel just below the “Dynamic Nomogram”. In the right of the screen shot, graphical summary, numerical summary, and model summary are all given. Users can select the items of each parameter, and finally click the “Predict” bottom.


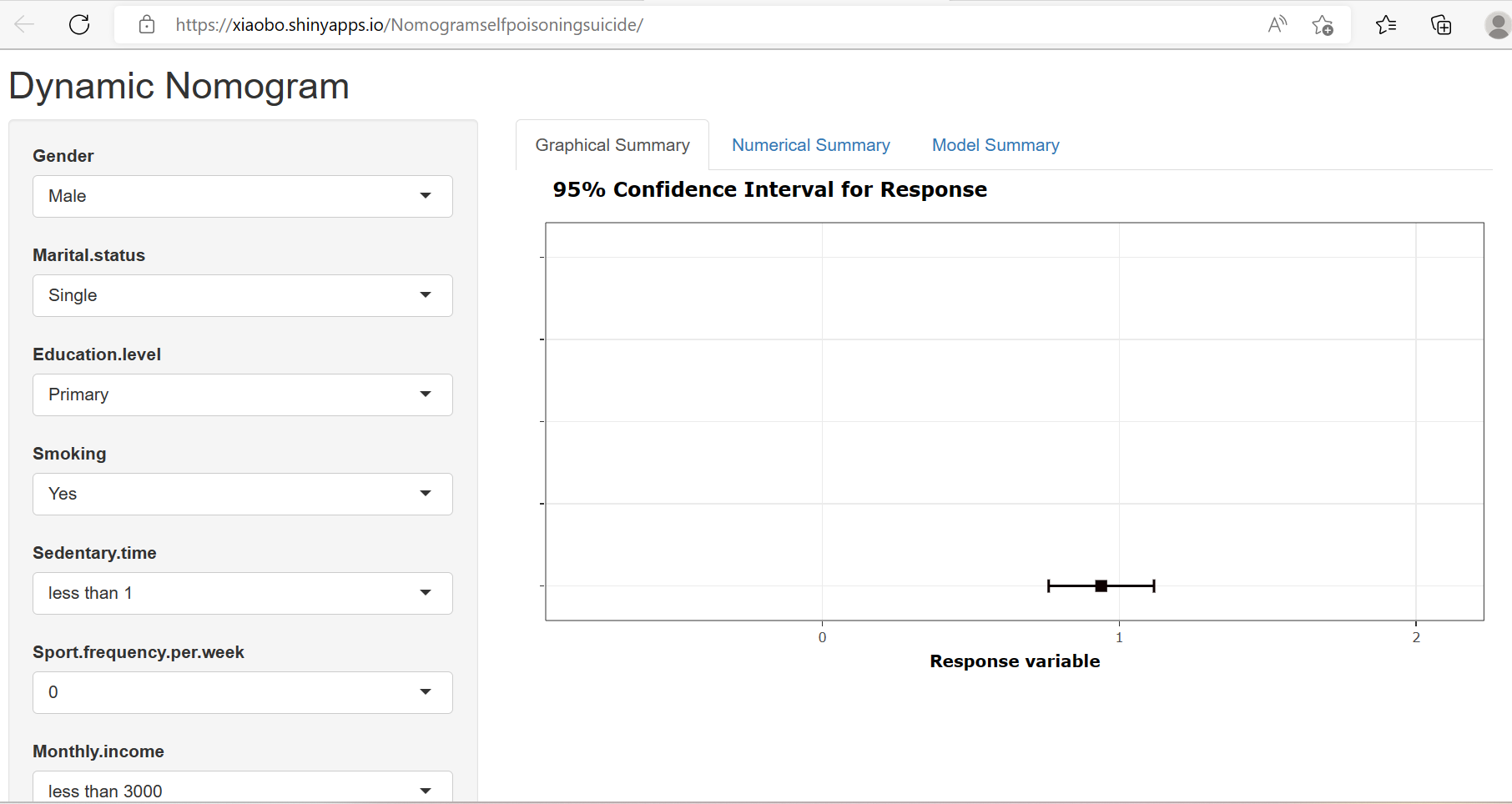


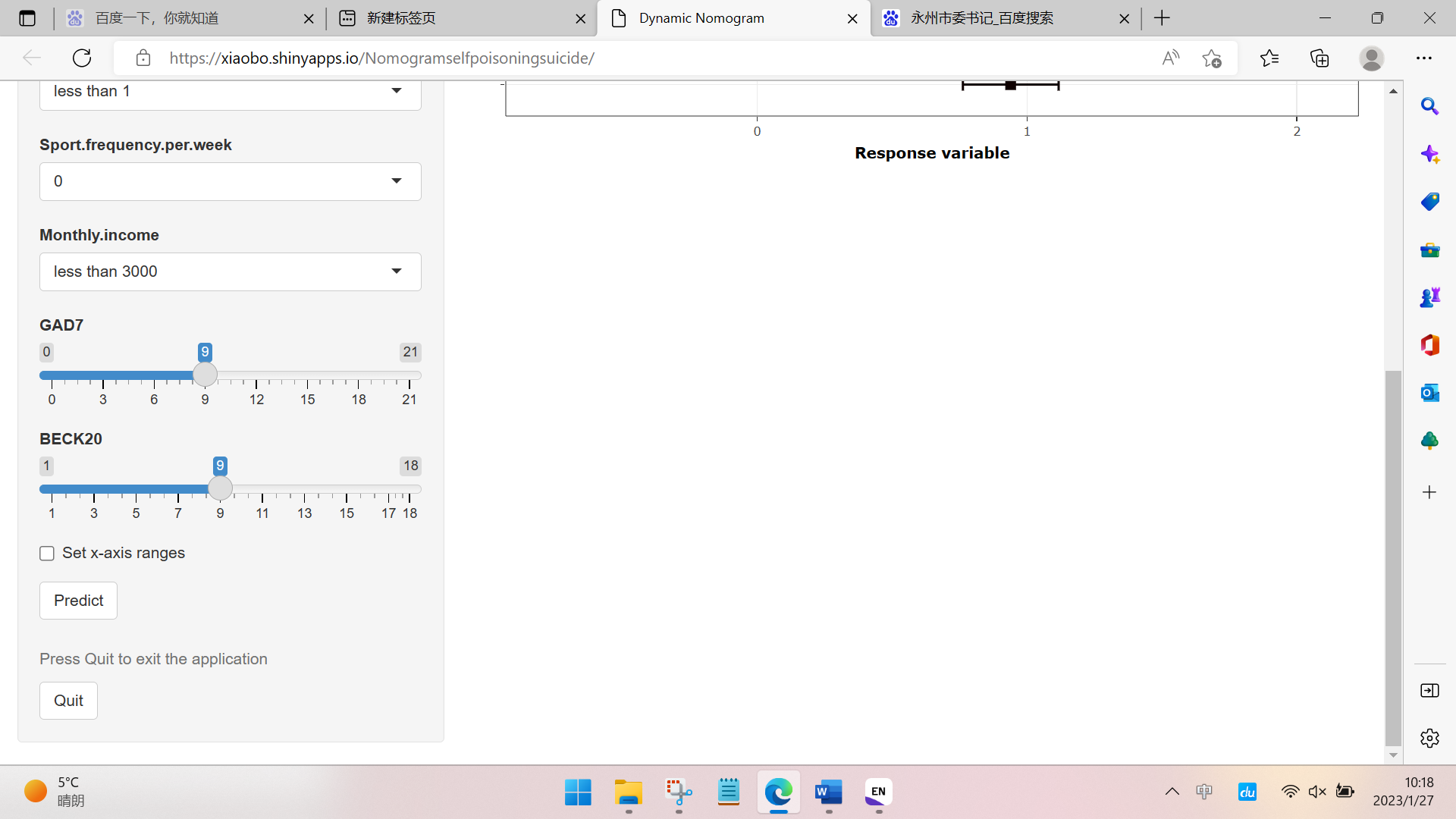


**2. Graphical summary**

In the summary, the predicted risk of suicide and corresponding 95% confidence interval for response are shown in a plot.


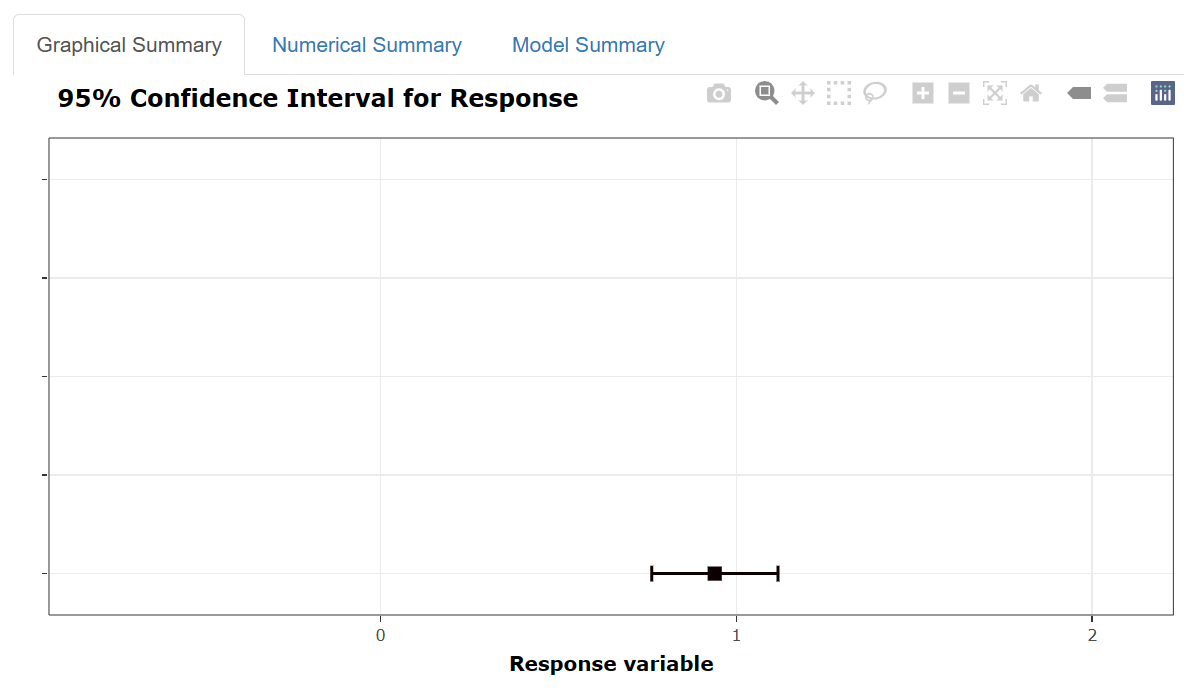


**3. Numerical summary**

In this summary, users can check the items of each model parameter. In addition, the detailed prediction risk (0.939) and corresponding 95% confidence interval (0.762-1.117) are shown.


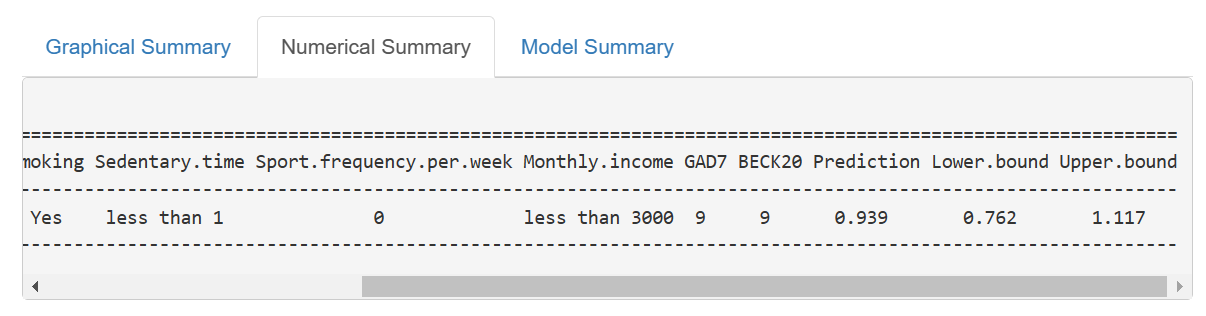


**4. Model summary**

In the summary, the model, deviance residuals, coefficients are all provided.


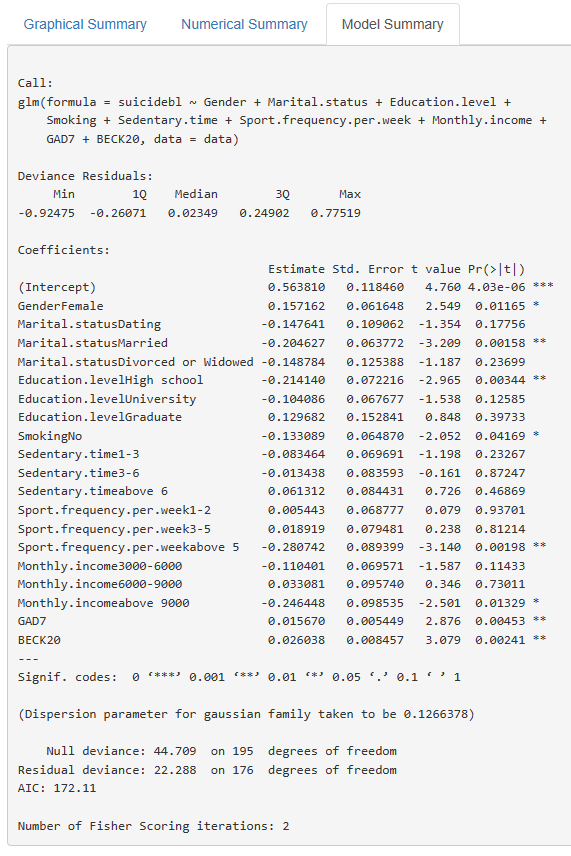

Supplement: Supplementary file 1 [file Table_1.DOCX]
